# Effect of early progressive mobilization on intensive care unit-acquired weakness in mechanically ventilated patients: An observational study

**DOI:** 10.1097/MD.0000000000031528

**Published:** 2022-11-04

**Authors:** Jing Zhou, Chao Zhang, Ji-dong Zhou, Cheng-kai Zhang

**Affiliations:** a Intensive Care Unit, The Fenghua People’s Hospital, Ningbo City, Zhejiang Province, China

**Keywords:** Critical illness, early progressive mobilization, intensive care unit-acquired weakness, mechanical ventilation, muscle strength

## Abstract

Early progressive mobilization is usually considered as an effective method for intensive care unit-acquired weakness (ICU-AW), but the controversies on this topic remain debatable, especially in initiation time, safety profile, and other implementation details. So, more studies should be performed to solve these disputes. A set of critically ill patients underwent mechanical ventilation in intensive care unit (ICU) of our hospital from March 2018 to September 2020 were included as study object. Patients received early progressive mobilization were included into the intervention group (n = 160), and another patients matched with the intervention group by gender, age, and APACHE II score, and these patients received routine intervention were included into the control group (n = 160). Then, indexes involving muscle strength, Barthel index, functional independence, incidence rates of ICU-AW and other complications were comparatively analyzed between the 2 groups. The Medical Research Council score and Barthel index score in the intervention group were significantly higher than those in the control group (all *P* < .05). The percentages of patients who were able to complete taking a shower, wearing clothes, eating, grooming, moving from bed to chair and using the toilet by alone in the intervention group were significantly higher than those in the control group (69.38% vs 49.38%, 73.13% vs 51.88%, 81.25% vs 55.63%, 74.38% vs 48.75%, 82.50% vs 65.63%, 78.13% vs 63.13%, respectively, all *P* < .05). The incidence rate of ICU-AW and overall incidence rate of complications in the intervention group were significantly lower than those in the control group (6.88% vs 28.13% and 23.13% vs 48.13%, both *P* < .05). Early progressive mobilization can effectively increase muscle strength and daily basic motion ability, improve functional status, and decrease risk of ICU-AW in critically ill patients underwent mechanical ventilation, and it has an attractive application value in clinic.

## 1. Introduction

Intensive care unit-acquired weakness (ICU-AW) is a common complication of severe disease, specifically occurring in intensive care unit (ICU) patients who undergo continuous mechanical ventilation, mainly characterized by limbs and trunk muscle force decline, weak tendon reflex, amyotrophia, and so on.^[1,2]^ It is estimated that exceeded 25% of patients with prolonged mechanical ventilation with more than a week may develop ICU-AW, which will lead to an obvious influence on clinical prognosis and quality of life, and parts of whom combined with sepsis and/or organ failure may result in an immediate life-threatening.^[3,4]^

At present, the exact pathogenesis of ICU-AW is not very clear yet, but the previous studies demonstrated that neuromuscular dysfunction caused by potential risk factors, such as systemic inflammation, glucocorticoid use, and long-term mechanical ventilation was a key contributor to the persisting functional disability.^[5]^ Over the past few years, therapeutic strategies for various clinical treatments have been described, including electrical stimulation, acupuncture, mobilization therapy, pharmacologic intervention and combination therapy. Among them, early progressive mobilization can significantly relieve neuromuscular dysfunction, promote muscle strength recovery, and played an important role in prevention and treatment of ICU-AW.^[6]^ However, there were great differences in implementation standards and methods in various reports, lacking of consistency on the clinical efficacy and implementation procedures in the current literatures. In this study, we aimed to investigate the effect of early progressive mobilization on ICU-AW in critically ill patients who underwent mechanical ventilation, so as to provide more references for clinical practice.

## 2. Materials and methods

### 2.1. Patients

**Figure 1.**
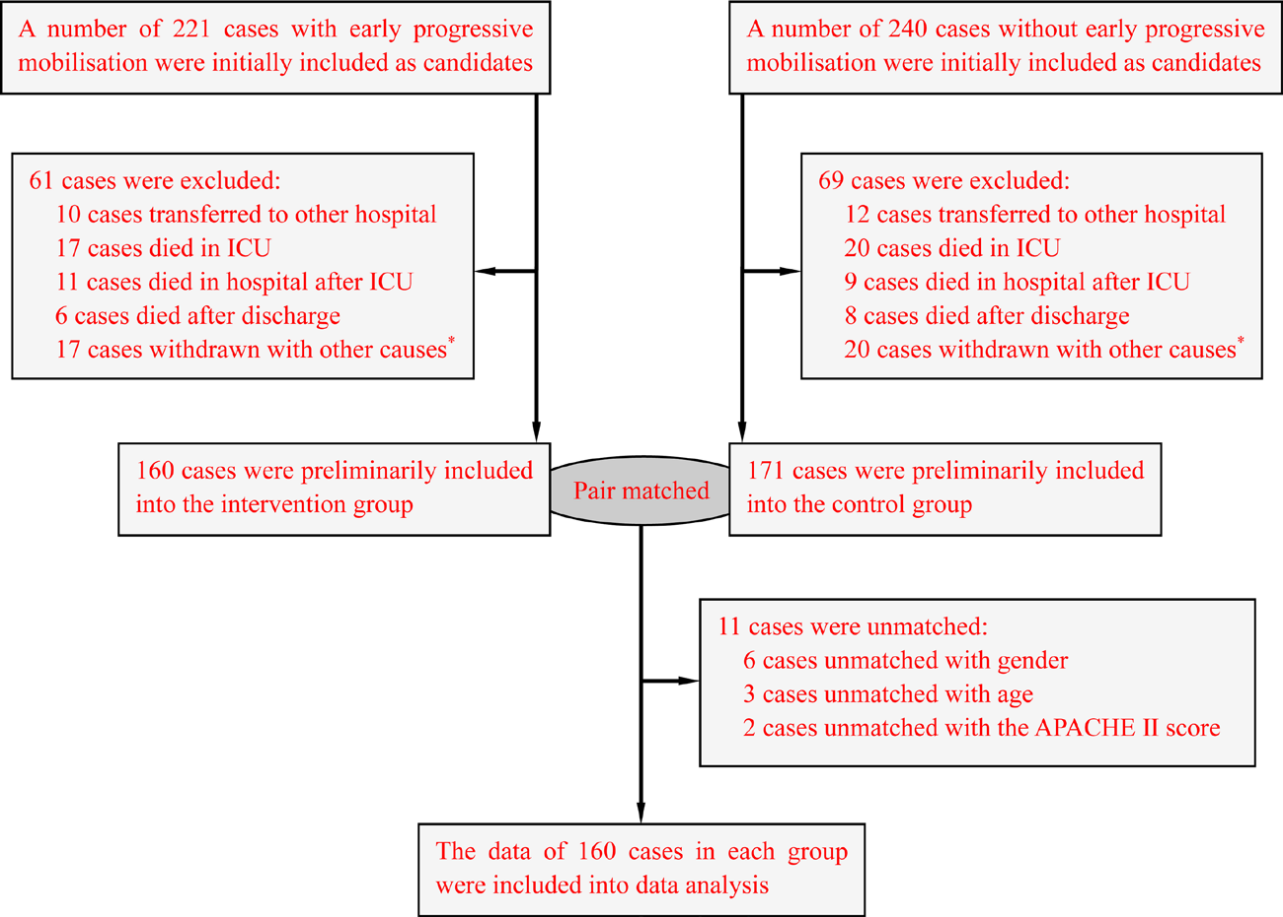
Flow chart of patients through the study. ^*^Other causes including data missing, patient or family members voluntarily requested discharge, not suitable for early progressive mobilization, etc.

From March 2018 to September 2020, a set of critical ill inpatients who underwent mechanical ventilation in ICU in our hospital were included into this study. Inclusion criteria included: patients with duration of mechanical ventilation greater than 72 hours; patients with a life expectancy of more than 6 months; patients who can accept early mobilization intervention with stable vital signs and hemodynamics. Exclusion criteria included: patients with a contraindication of early progressive mobilization; patients with previous history of neuromuscular disease, terminal cancer, or other extremely poor prognosis; patients transferred to other hospitals, died or withdrawn with other causes. Then, these eligible patients underwent early progressive mobilization were assigned to the intervention group (routine intervention combined with early progressive mobilization, n = 160). Meanwhile, another patients matched in 1:1 ratio with the intervention group by gender, age (±5 years), and acute physiology and chronic health evaluation II (APACHE II) score (±3 points), and those patients received routine intervention were included into the control group (routine intervention, n = 160). The data of all included patients were collected, and a matched case-control study was performed to comparatively analyze differences of the 2 schemes. The flow chart of patients through the study was shown as in Figure 1.

### 2.2. Methods

All patients received the routine therapy and management of ICU. Patients in the control group received routine treatment and nursing, involving vital signs monitoring, tubes and ventilator care, position management, nutritional support, and other symptomatic and supportive treatments. Patients in the intervention group received early progressive mobilization combined with the routine intervention. It consisted of these items: (1) Assessment of risk: Patient’s tolerance and risk were evaluated before each mobilization, and a duty doctor chose passive mobilization or active exercise based on patient’s disease type, conscious status, and muscle strength; (2) Implementation process: Firstly, during the unconscious phase, passive mobilization such as turning over, massage, flexion, and extension of limb joints were performed daily 3 times, and repeated no less than 10 times; Secondly, during the consciousness recovering phase, active exercise coupled with passive mobilization was performed daily 3 times, flexion and extension of joint no less than 10 times, and encouraging patients to engage in active joint exercise, performed daily 3 times with no less than 5 times in each time; Thirdly, during fully conscious phase, active exercises such as turning over, eating, drinking, putting on or taking off clothes in the bed were performed, doing resistance exercises from gently to hard daily 3 times, no less than 10 times in each time; Lastly, during the bed-leaving phase, active exercise such as standing and sitting, gait exercise and walking were given under the protection and experienced more than 2 weeks, the frequency of exercise increased or decreased based on patient’s conditions; (3) Intervention initiation, suspension, and termination: early progressive mobilization was starting within 24 to 72 hours after mechanical ventilation when patients meet implementation requirements; If abnormal vital signs (systolic blood pressure < 80 or > 180 mm Hg, heart rate < 50 or > 120 times per min, respiratory rate < 15 or > 30 times/min) and other high risk condition (including persistent elevated intracranial pressure, acute myocardial ischemia, gastrointestinal bleeding, and so on) occurred, the intervention was suspended, and the suspended intervention can be gradually restarted when the patient’s condition was stable. When a patient had achieved a well functional status, the intervention would be terminated.

### 2.3. Study indexes

Baseline data, muscle strength measurement and Barthel index score before and after intervention, and the functional independence, incidence of ICU-AW, and other complications during observation period were recorded. Muscle strength was measured by the Medical Research Council score (MRC score), which consisted of 12 items, with 0 to 5 points per item. The higher the score, the stronger the muscle strength. When MRC score was less than 48 points, ICU-AW was identified.^[7]^ The Barthel index score involved 10 items, such as eating, taking a shower, wearing clothes, etc. with a total score of 100. A higher score indicated a higher self-care ability.^[8]^ The functional independence assessment included 6 skills: taking a shower, wearing clothes, eating, grooming, moving from bed to chair, and using the toilet. The patients with an ability of independent completion for above items was considered as a well functional independence.^[9]^

### 2.4. Statistical analysis

Statistical analysis of data was performed by using SPSS 22.0 software. The measurement data were presented as mean ± standard deviation (^–^*x* ± s), grouped *t* test was used for the comparison between the 2 groups, paired *t* test was used for the intragroup comparison before and after intervention, and the Shapiro–Wilk test was used to test for normality of data distribution. The categorical data were presented as frequency and percentage (%), analyzed by *x*^*2*^ test or *x*^*2*^ test with Yates’s correction. *P* < .05 was considered as significance in statistics.

## 3. Results

### 3.1. Comparison of baseline data

For the comparison of baseline data, including gender, age, body mass index, APACHE II score, mechanical ventilation time, length of ICU stay, length of hospital stay, medications use, primary disease, treatment strategy, and comorbidities, no significant differences were observed (*P* > .05) between the 2 groups, as shown in Table 1.

**Table 1:** Comparison of baseline data between the 2 groups [(n/%), (^–^x ± s)].

Indexes	Intervention group (n=160)	Control group (n=160)	*t/x* ^*2*^	*P*
Gender (n)				
Male	84 (52.50)	84 (52.50)	-	1.000
Female	76 (47.50)	76 (47.50)		
Age (yr)	60.78 ± 8.45	61.40 ± 7.29	0.703	.483
BMI (kg/m^2^)	26.45 ± 4.26	25.94 ± 3.71	1.142	.254
APACHE II score (point)	16.85 ± 4.72	17.16 ± 5.08	0.565	.572
Mechanical ventilation time (d)	7.31 ± 2.36	8.56 ± 2.57	1.611	.108
Length of ICU stay (d)	14.89 ± 4.62	15.07 ± 4.97	0.336	.737
Length of hospital stay (d)	25.89 ± 8.54	27.23 ± 9.17	1.353	.177
Medications use^*^ (n)	146 (91.25)	139 (86.88)	1.572	.210
Primary disease type (n)				
Cardia-cerebrovascular diseases	46 (28.75)	49 (30.63)	0.135	.714
Gastrointestinal diseases	40 (25.00)	36 (22.50)	0.276	.599
Respiratory diseases	24 (15.000)	31 (19.38)	1.076	.300
Hepatic/renal/bladder diseases	23 (14.38)	19 (11.88)	0.439	.508
Others	27 (16.88)	25 (15.63)	0.092	.762
Treatment strategy (n)				
Conservative treatment	95 (59.38)	101 (63.13)	0.474	.491
Operative treatment	65 (40.63)	59 (36.88)		
Comorbidities (n)				
Hypertension	53 (33.13)	44 (27.50)	1.198	.274
Diabetes	28 (17.50)	30 (18.75)	0.084	.772
Coronary heart disease	27 (16.88)	22(13.75)	0.602	.438
Chronic bronchitis	15 (9.38)	18 (11.25)	0.304	.581
Others	24 (15.00)	29 (18.13)	0.565	.452

ICU = intensive care unit.

* Including corticosteroids, neuromuscular blockers, vasopressors, and so on.

### 3.2. Comparison of MRC score and Barthel index score

The MRC score and Barthel index score were significantly increased after intervention than before (*P* < .05), and the scores of intervention group were significantly higher than those of the control group (*P* < .05), as shown in Table 2.

**Table 2:** Comparison of MRC score and Barthel index score in the 2 groups (^–^x ± s).

Indexes		Intervention group (n=160)	Control group (n=160)	*t*	*P*
MRC score	Before intervention	46.22 ± 4.36	46.44 ± 4.77	0.431	.667
	After intervention	57.35 ± 4.18	48.51 ± 5.38	16.412	<.001
	*t*	23.309	3.642		
	*P*	<0.001	<0.001		
Barthel index score	Before intervention	67.74 ± 5.33	68.38 ± 5.69	1.038	.300
	After intervention	83.26 ± 6.21	70.49 ± 6.82	17.512	<.001
	*t*	23.988	3.005		
	*P*	<0.001	0.003		

MRC score = Medical Research Council score.

### 3.3. Comparison of functional independence

After the intervention, the functional independence of taking a shower, wearing clothes, eating, grooming, moving from bed to chair, and using the toilet of the intervention group were higher than those of the control group, the differences were statistically significant between the 2 groups (*P* < .05), as shown in Table 3.

**Table 3:** Comparison of functional independence between the 2 groups (n/%).

Indexes	Intervention group (n=160)	Control group (n=160)	*x* ^*2*^	*P*
taking a shower	111 (69.38)	79 (49.38)	13.266	<.001
Wearing clothes	117 (73.13)	83 (51.88)	15.413	<.001
Eating	130 (81.25)	89 (55.63)	24.319	<.001
Grooming	119 (74.38)	78 (48.75)	22.200	<.001
Moving from bed to chair	132 (82.50)	105 (65.63)	11.859	.001
Using the toilet	125 (78.13)	101 (63.13)	8.676	.003

### 3.4. Comparison of incidences of ICU-AW and other complications

The incidence of ICU-AW and the overall incidence rate of the intervention group were significantly higher than those of the control group (*P* < .05), but there was no significant differences in the incidence of other single complication between the 2 groups (*P* > .05). As shown in Table 4.

**Table 4:** Comparison of ICU-AW and complications between the 2 groups (n/%).

Indexes	Intervention group (n=160)	Control group (n=160)	*x* ^*2*^	*P*
ICU-AW	11 (6.88)	45 (28.13)	25.022	<.001
Ventilator-associated pneumonia	14 (8.75)	11 (6.88)	0.391	.532
Pressure ulcer	2 (1.25)	7 (4.38)	1.829	.176^*^
Deep vein thrombosis	3 (1.88)	5 (3.13)	0.128	.720^*^
Stress gastrointestinal bleeding	2 (1.25)	4 (2.50)	0.170	.680^*^
Others	5 (3.13)	6(3.75)	0.094	.759
Overall incidence rate	37 (23.13)	77 (48.13)	21.802	<.001

ICU-AW = intensive care unit-acquired weakness.

* *x*^*2*^ test with Yates’s correction.

## 4. Discussion

Traditional ICU medical care mainly focuses on the maintenance of the functions of vital organs such as the heart, lung, liver, and kidney, to ensure the life safety of critical illness survivors. In recent years, ICU-AW has gradually become a hotpot in the field of critical care medicine, various intervention schemes for ICU-AW have also attracted more and more researchers’ attentions. some studies suggested that ICU-AW may occur within several hours after patients receiving mechanical ventilation, the early intervention can reduce incidence of ICU-AW, and the earlier intervention can achieve the better effect.^[10–12]^ So it has an important clinical value to explore an optimum proposal for early progressive mobilization.^[13]^

In this study, The MRC score and Barthel index score of the intervention group were significantly higher than those of the control group, suggesting that early progressive mobilization can effectively improve the muscle weakness. As we known, due to the sedative effect, mechanical ventilation patients are commonly unconscious and inactive for a long time, making it easy to develop a time-dependent decline in muscle strength.^[14,15]^ Those patients of the intervention group receiving early progressive mobilization from passive movement to active exercise, effectively activated extension-contraction cycle of joints and maintained muscle flexibility by turning over, massage, and passive flexion and extension of limb joints, and other treatments, subsequently increased self-care ability of patients. This result was consistent with the report of Wu H et al^[16]^ Furthermore, the proportion of patients who can independently complete daily actions (eating, taking a shower, wearing clothes, grooming, moving from bed to chair, using the toilet) in the intervention group were significantly higher than those of the control group, indicating that in the case of ensuring safety, early progressive mobilization can help patients to recover functional independence, which was consistent with the report of Schaller SJ et al^[17]^

For safety reasons, early progressive mobilization was generally not recommended for ICU inpatients in the past. In this study, early progressive mobilization was implemented after assessment of risk. After that, no obvious differences were observed in the mortality of patients in ICU, hospital after ICU, and post-discharge, but the incidence of ICU-AW and overall complication rate in patients of the intervention group were significantly lower than those of the control group. These results mentioned above suggested that early progressive mobilization was a feasible and safe protocol in the prevention and treatment of ICU-AW, and this viewpoint in our study was supported by other study from Piva TC et al^[18]^ According to current reports, the mechanism by which early progressive mobilization reduces the incidence of ICU-AW and other complications remain unclear. Some studies have found that the levels of various inflammatory mediators in muscle of ICU-AW patients are significantly higher than those of patients without ICU-AW, speculated which may be associated with the reduction of inflammatory response by early progressive mobilization.^[19,20]^ There was also a viewpoint that massage and joint exercise for patients can improve blood circulation and metabolism of muscle tissue, inhibit myohemoglobin decline and relieve neuromuscular dysfunction of patients, thereby reducing the risk of ICU-AW and other complications.^[21,22]^

At present, the current implementing protocol of ICU-AW was still in the exploratory stage. So it is necessary to make further exploration in practice to solve these disputes in the security, implementation details, and other aspects. For example, there was no consensus the initiation time of early progressive mobilization. A meta-analysis pointed out that the early progressive mobilization should be performed within 72 hours after mechanical ventilation.^[23]^ In this study, the initiation time of early progressive mobilization started within 24 to 72 hours when patients met implementation requirements. Our results showed that early progressive mobilization within 24 to 72 hours had some favorable clinical outcomes comparing with the control group. However, there is a deficiency in description and comparison regarding the differences among patients with various initiation time at 24, 48, or 72 hours after mechanical ventilation, when is the optimum time for initiation of early progressive mobilization needs to be further explored in future study. What is more, our study belongs to a case-control study with pair matched, and these results require to be confirmed in multicentre randomized controlled trials.

In conclusion, early progressive mobilization could effectively increase muscle strength and daily basic motion ability, improve functional status, and decrease incidence of ICU-AW in critically ill patients who underwent mechanical ventilation. Under the condition of ensuring the safety of patients, early progressive mobilization has an attractive application value, which should be promoted in prevention and treatment of ICU-AW.

## Author contributions

**Conceptualization:** Jing Zhou, Ji-dong Zhou.

**Data curation:** Jing Zhou, Chao Zhang.

**Formal analysis:** Cheng-kai Zhang.

**Supervision:** Ji-dong Zhou.

**Writing—original draft:** Jing Zhou.

**Writing—review and editing:** Chao Zhang, Cheng-kai Zhang.
